# Opportunities involving microfluidics and 3D culture systems to the *in vitro* embryo production

**DOI:** 10.1590/1984-3143-AR2023-0058

**Published:** 2023-08-04

**Authors:** Marcia de Almeida Monteiro Melo Ferraz, Giuliana de Avila Ferronato

**Affiliations:** 1 Faculty of Veterinary Medicine, Ludwig-Maximilians University of Munich, Oberschleißheim, Germany; 2 Gene Center, Ludwig-Maximilians University of Munich, Munich, Germany

**Keywords:** bioprinting, microfluidic, embryo development, biotechnologies

## Abstract

Traditional methods of gamete handling, fertilization, and embryo culture often face limitations in efficiency, consistency, and the ability to closely mimic *in vivo* conditions. This review explores the opportunities presented by microfluidic and 3D culture systems in overcoming these challenges and enhancing *in vitro* embryo production. We discuss the basic principles of microfluidics, emphasizing their inherent advantages such as precise control of fluid flow, reduced reagent consumption, and high-throughput capabilities. Furthermore, we delve into microfluidic devices designed for gamete manipulation, *in vitro* fertilization, and embryo culture, highlighting innovations such as droplet-based microfluidics and on-chip monitoring. Next, we explore the integration of 3D culture systems, including the use of biomimetic scaffolds and organ-on-a-chip platforms, with a particular focus on the oviduct-on-a-chip. Finally, we discuss the potential of these advanced systems to improve embryo production outcomes and advance our understanding of early embryo development. By leveraging the unique capabilities of microfluidics and 3D culture systems, we foresee significant advancements in the efficiency, effectiveness, and clinical success of *in vitro* embryo production.

## Introduction

*In vitro* embryo production (IVEP) has become an indispensable tool in various fields, including assisted reproductive technologies (ARTs), conservation of endangered species (Comizzoli, 2015), and fundamental research in early embryonic development. Despite the significant advancements in IVEP, several challenges persist, such as low efficiency in embryo production, variability in embryo quality, and the inadequacy of existing *in vitro* models to accurately mimic *in vivo* conditions (Menezo et al., 2018). To overcome these obstacles and improve IVEP outcomes, researchers have turned to emerging technologies, such as microfluidics and 3D culture systems (Esteves et al., 2013; Ferraz et al., 2018b, 2017; Kieslinger et al., 2015). These novel approaches hold great promise in addressing the limitations of conventional IVEP methods and revolutionizing the field of reproductive biology.

Microfluidics is a rapidly growing field that involves the manipulation of small volumes of fluids in microscale channels, offering precise control over the cellular microenvironment (Whitesides, 2006), superior nutrient and waste exchange (Berthier et al., 2012), and the potential for high-throughput screening (Sackmann et al., 2014). This technology has been increasingly applied to various aspects of IVEP, including oocyte and sperm handling (Beckham et al., 2018; Wagenaar et al., 2015; El-Sherry et al., 2014; Scherr et al., 2015), *in vitro* fertilization (IVF) (Clark et al., 2005; Huang et al., 2018; Suh et al., 2006), and embryo culture and assessment (Esteves et al., 2013; Huang et al., 2015; Karcz et al., 2023; Kieslinger et al., 2015; Kim et al., 2009). 3D culture systems, on the other hand, provide a more physiologically relevant environment for cell growth and differentiation compared to traditional 2D culture systems (Baker and Chen, 2012). By mimicking the *in vivo* extracellular matrix (ECM), 3D culture systems allow for improved cell-to-cell and cell-to-ECM interactions (Nicolas et al., 2020), thereby enhancing cell development and functionality. Furthermore, these systems offer valuable insights into embryo-maternal communication and the role of the microenvironment in early development (Ferraz et al., 2018b, 2017).

This review aims to provide a comprehensive overview of the current state of microfluidics and 3D culture systems in the context of IVEP, including their applications, challenges, and limitations. Moreover, the integration of microfluidics and 3D culture systems will be explored, highlighting the potential benefits of combining these two technologies for *in vitro* embryo production. Finally, the future perspectives and potential impact of microfluidics and 3D culture systems in reproductive medicine and basic research will be discussed. By assessing the latest advancements and challenges in microfluidics and 3D culture systems for IVEP, this review seeks to contribute to the ongoing development of more efficient, and reliable approaches to *in vitro* embryo production.

## Use of microfluidic for gametes and embryo manipulation

### Basic principles of microfluidics

Microfluidics is a multidisciplinary field that involves the manipulation, control, and analysis of small volumes of fluids, typically in the range of microliters to picoliters, within microscale channels and devices (Whitesides, 2006). The key concepts of microfluidics include laminar flow, which refers to the smooth and orderly movement of fluid in parallel layers without turbulence; high surface-to-volume ratios, which enable rapid heat and mass transfer; and precise control over fluid dynamics (Squires and Quake, 2005). This precise control is achieved using microscale features such as channels, chambers, and valves, as well as the application of external forces such as pressure, electric fields, and magnetic fields to manipulate fluid flow. Microfluidic systems offer several advantages that make them particularly suitable for applications in IVEP, including:

- *Precise control of fluid flow and the local microenvironment:* Microfluidic devices allow for the precise manipulation of fluid flow rates, fluid mixing, and the introduction of chemical gradients (Sackmann et al., 2014; Squires and Quake, 2005; Whitesides, 2006), enabling the fine-tuning of the culture environment to optimize embryo development.
- *Reduced sample and reagent volumes:* Due to their small dimensions, microfluidic devices require significantly smaller volumes of samples and reagents compared to conventional macro-scale systems (Sackmann et al., 2014; Squires and Quake, 2005; Whitesides, 2006). This reduction in volume not only minimizes reagent costs but also limits the potential for detrimental effects of reagent toxicity on embryo development.
- *Rapid mixing and diffusion:* The high surface-to-volume ratios in microfluidic devices enable rapid mixing and diffusion of solutes, which allows for efficient nutrient delivery and waste removal (Sackmann et al., 2014; Squires and Quake, 2005; Whitesides, 2006), both of which are critical factors for maintaining optimal embryo development.
- *High-throughput and parallel processing:* Microfluidic devices can be designed to process multiple samples simultaneously, enabling high-throughput screening and analysis (Sackmann et al., 2014). This feature is particularly useful in IVEP, where the identification of high-quality embryos is essential for improving clinical outcomes.
- *Biocompatibility and optical transparency:* Microfluidic devices can be fabricated from biocompatible materials such as polydimethylsiloxane (PDMS), which are non-toxic, optically transparent, and gas permeable (Berthier et al., 2012; Ferraz et al., 2018a; Ferraz et al., 2020a). The optical transparency enables real-time, non-invasive imaging of embryos, while gas permeability ensures adequate oxygen and carbon dioxide exchange.

Microfluidic devices often incorporate microchannels and microvalves to manipulate fluid flow and control the local environment. Microchannels, which are narrow conduits with dimensions in the micrometer range, confine fluid flow and can be designed with various geometries to control fluid mixing, create gradients, or direct the movement of particles or cells (Beebe et al., 2002, 2000). Microvalves, on the other hand, are small-scale flow control elements that can be actuated using pneumatic, mechanical, or electrostatic forces, allowing for precise control of fluid flow rates and direction within the device (Beebe et al., 2000). The integration of these components could enable the manipulation of gametes and embryos in a controlled and gentle manner, reducing the risk of mechanical stress or damage to gametes and embryos during processes such as oocyte maturation, IVF, and embryo culture.

### Oocyte handling, maturation and cryopreservation

**Figure 1:**
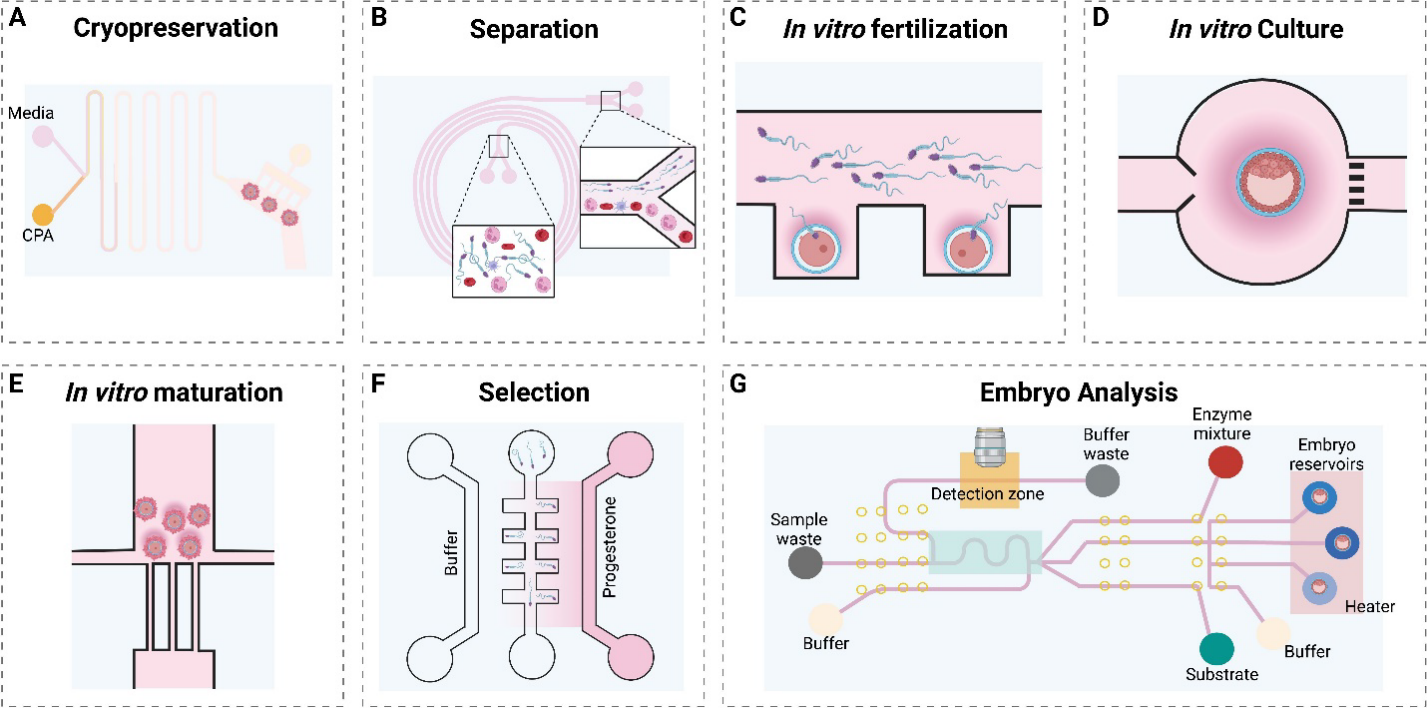
Microfluidics models for gametes and embryo handling and analysis. In (**A**) on chip oocyte CPA exposure model proposed by (Lai et al., 2015). In (**B**) design of a microfluidic platform for sperm separation from blood cells (Son et al., 2017). A flow free device that integrates a chemical gradient for sperm selection by chemotaxis is shown in (**F**) (Berendsen et al., 2020). In (**E**) oocyte maturation device adapted from (Berenguel-Alonso et al., 2017). *In vitro* fertilization prototypes were also designed (**C**) (Han et al., 2010). Microfluidic platforms for embryo culture (**D**) and on chip metabolite analysis (**G**) were also produced (Esteves et al., 2013; Heo et al., 2012). Figure made with Biorender.

In the context of oocytes, microfluidic offer a platform for automated manipulation and potentially high-throughput environment for various processes, including the removal of cumulus cells, oocyte maturation, and exposure to media gradients, such as cryoprotectants. These platforms integrate specialized structures and sensors to achieve optimal results. For instance, cumulus cells can be effectively removed using integrated microstructures designed to gently separate the cells from the oocyte without causing damage (Zeringue et al., 2005). Microfluidic platforms also facilitate the maturation of oocytes by continuously exposing them to the maturation medium (Berenguel-Alonso et al., 2017) (Figure 1E), ensuring that the optimal conditions for maturation are maintained throughout the process. Moreover, microfluidic platforms could allow for the selection of oocytes based on their maturation level and quality by incorporating integrated optical or mechanical sensors (Yanez and Camarillo, 2017). These sensors have the potential to assess parameters such as oocyte size, shape, and zona pellucida thickness, which can be indicative of oocyte quality and maturity. Additionally, microfluidic devices can be designed to mimic the *in vivo* environment by incorporating co-culture with cumulus cells, granulosa cells, or other supporting cells to facilitate oocyte maturation and improve oocyte quality.

Microfluidics also holds the potential to revolutionize oocyte cryopreservation by automating the processes of cryoprotectant agent (CPA) loading and removal, ensuring precise exposure timing, and reducing osmotic and thermal stress. This technology allows for real-time monitoring of cellular responses, such as membrane permeability and volume changes, while evaluating various exposure scenarios. In these devices, cells are individually trapped in dedicated microstructures (Heo et al., 2011) or chambers (Guo et al., 2019), and CPA mixtures are prepared on-chip at the desired concentration. The mixtures are loaded into the exposure chamber (Guo et al., 2019; Heo et al., 2011) using stepwise concentration changes, linear gradients, or more complex patterns (Heo et al., 2011; Lai et al., 2015). In a more sophisticated setup, CPAs are mixed *in situ* using discrete droplets and electrowetting-on-dielectric (EWOD) technology. This approach allowed for automated, stepwise delivery to individual mouse embryos isolated in sub-microliter droplets, with no adverse effects on embryo survival and developmental rates after vitrification (Pyne et al., 2014). When using microfluidics, murine and bovine oocytes and zygotes experienced less shrinkage, exhibited better morphology, and demonstrated higher cell quality and improved developmental competence (Lai et al., 2015) (Figure 1A). A stepwise unloading of CPAs on porcine oocytes has also been shown to improve survival, embryo cleavage, and blastocyst formation (Guo et al., 2019).

Overall, the use of microfluidic platforms in oocyte handling and processing offers numerous advantages over traditional methods, including reduced stress on the oocytes, higher throughput, and improved selection accuracy. The integration of specialized structures and sensors within these platforms further enhances their potential to advance reproductive biology and optimize fertility treatments.

### Sperm selection and capacitation

In the female reproductive tract, sperm sorting occurs through various mechanisms to ensure that only the most viable and competent sperm cells have the opportunity to fertilize the oocyte. Some examples of sperm sorting mechanisms in the female reproductive tract include: (1) Cervical mucus, which acts as a selective barrier that only allows the passage of motile and morphologically normal sperm, filtering out abnormal or immotile sperm, as well as potential contaminants such as bacteria or cellular debris (Suarez, 2016); (2) Sperm reservoirs which are present in the oviducts, specifically the isthmus, selectively bind and store sperm, releasing them gradually over time to prolong their presence in the oviduct and increase the likelihood of successful fertilization (Pollard et al., 1991); (3) Immune response, the female reproductive tract has an immune system that can identify and eliminate sperm cells with damaged or abnormal structures, ensuring that only the healthiest sperm cells have the opportunity to fertilize the oocyte (Robertson, 2005); (4) Sperm capacitation, as sperm cells travel through the female reproductive tract, they undergo capacitation, which prepares them for fertilization (Suarez, 2016); and (5) Chemotaxis, studies suggest that sperm cells are guided towards the oocyte by chemical gradients released by the oocyte itself or by the surrounding cells, this chemotactic guidance helps to direct the most motile and responsive sperm cells towards the oocyte, increasing the likelihood of successful fertilization (Suarez, 2016).

Drawing inspiration from natural sperm sorting mechanisms, microfluidic-based sperm selection methods have been developed to mimic *in vivo* conditions and enhance ART outcomes. These innovative techniques focus on sorting sperm based on factors such as motility, morphology, and DNA integrity to improve the chances of successful fertilization. Holographic imaging of sperm cells trapped in microfluidics channels, has been used for sperm selection. This method captures the 3D structure of sperm cells, allowing for the assessment of morphology and motility patterns in real-time (Di Caprio et al., 2015). Passive techniques, such as the use of microchannels and filters, have been effectively employed to separate sperm from white blood cells and other debris by exploiting size differences between the cells (Berendsen et al., 2020, 2019; Son et al., 2017) (Figure 1B). Additionally, sperm selection and analysis have been performed based on various criteria. For example, chemotactic response sorting utilizes chemical gradients to attract and select sperm cells that exhibit strong chemotactic behavior (Berendsen et al., 2020) (Figure 1F), while thermotactic behavior sorting leverages sperm cells' ability to sense and respond to temperature gradients to guide them toward the oocyte (Ko et al., 2018). Moreover, some methods combine multiple factors, such as motility, chemotaxis, and thermotaxis, for a more comprehensive selection approach (Xie et al., 2010).

The consistent production of high-quality, motile sperm with undamaged DNA in humans (Parrella et al., 2018) has been made possible through the development and application of advanced microfluidic-based sperm selection methods, which have also led to improvements in cattle insemination success rates (Nagata et al., 2018) and increased pregnancy rates following intracytoplasmic sperm injection (ICSI) in humans (Parrella et al., 2018). These microfluidic sperm selection methods offer several advantages over traditional techniques, such as the swim-up method and density gradient centrifugation. For instance, microfluidic methods are less invasive, do not require the use of potentially harmful chemicals, and can be more easily automated and standardized. Additionally, they can be integrated with other laboratory processes, such as the culturing of oocytes or embryos, further streamlining the ART workflow.

### *In vitro* fertilization, embryo culture and analysis

Microfluidic systems have emerged as a promising technology for IVF due to their potential to improve the efficiency and accuracy of fertilization and embryo incubation while reducing human intervention and variability. Microfluidic devices can be designed to bring oocytes and sperm into close proximity under controlled conditions, which can improve fertilization rates compared to conventional static co-incubation methods.

A microwell array to simplify oocyte handling and manipulation, allowing rapid and convenient medium changing, and enabling automated tracking of single-embryo development was created and yielded a comparable fertilization rate to the conventional microdrop-in-a-dish technique (Han et al., 2010) (Figure 1C). Clark et al. used a geometrical constraint to block the porcine oocyte within a microfluidic chamber, which allowed the sperm to swim towards the oocyte but prevented them from penetrating it, resulting in a lower incidence of polyspermy (Clark et al., 2005). A microfluidic device that integrated each step of IVF, including oocyte positioning, sperm screening, fertilization, medium replacement, and embryo culture allowed efficient motile sperm selection and facilitated rapid medium replacement (Ma et al., 2011). This study showed that the average mouse sperm motility increased from 60.8% to 96.1% by passing through the screening channels. The embryo growth rate and blastocyst formation were similar between the microfluidics group and the conventional microdrop group (Ma et al., 2011).

Another microfluidic system featured a barrier gate that blocked the oocyte without deformation and allowed the selective passage of sperm, resulting in an increase in local sperm concentration around the trapped oocyte (Suh et al., 2006). They showed that a smaller total number of sperm were required to achieve similar fertilization rates compared to the conventional technique. A microfluidic *in vitro* culture system for bovine embryos, in which peristaltic muscle contraction was mimicked by using a partially constricted channel generated a gravity-driven dynamic flow using a micro-modulated tilting machine, resulting in a higher proportion of eight-cell development compared to a straight channel (Kim et al., 2009).

Heo et al. developed an embryo culture and assay device to perform automated periodic analysis of embryo metabolism. The microfluidic system represented a compelling case for integrated microfluidic platforms to perform practical single-embryo culture and real-time biochemical analysis, which has the potential of improving the success in clinical ART laboratories by screening high-quality embryos (Heo et al., 2012) (Figure 1G). A microfluidic device powered by EWOD has been developed to culture mouse embryos in a single droplet in a microfluidic environment. The dynamic culture using EWOD technology has been found to significantly increase the rate of embryo cleavage to a hatching blastocyst compared to traditional static culture, and transferring the embryos to pseudo-pregnant female mice has resulted in live births (Huang et al., 2015).

Overall, these studies demonstrate the potential of microfluidic systems in improving different stages of IVF and embryo culture, with the potential to simplify oocyte handling, reduce the required number and concentration of sperm, improve the fertilization rate, and enable real-time biochemical analysis of embryos. The optical transparency and gas permeability of microfluidic devices can enable real-time, non-invasive imaging and monitoring of embryo development (Esteves et al., 2013; Khalili and Rezai, 2019; Kieslinger et al., 2015). Time-lapse imaging can be combined with automated image analysis and machine learning algorithms to assess morphological and developmental parameters, such as cell division timing and blastocyst formation, which can be used to predict embryo quality and implantation potential (Lemmen et al., 2008). Moreover, microfluidic devices can be designed to accommodate multiple embryos simultaneously, enabling high-throughput screening and analysis (Sackmann et al., 2014). By providing real-time feedback on embryo development and metabolic activity, microfluidic systems have the potential to facilitate more informed decision-making during embryo selection, potentially improving clinical outcomes in ARTs.

## Use of 3D cultures for gametes and embryo manipulation

In 1912, Carrel described 3D cell culture as an alternative to traditional 2D culture systems (Carrel, 1912). Unlike 2D culture systems, where cells attach to a flat surface such as a petri dish, 3D culture allows cells to maintain their original morphology, which may promote more physiologically relevant cell proliferation and differentiation (Jensen and Teng, 2020). As the name already says, 3D culture allows cells to interact in their three dimensions, which improves its contact both with the environment and with other cells around it (Hoffman, 2018). To obtain a 3D culture system, different types of matrices and materials can be used, from very simple techniques that only prevent cells to adhere to the bottom of the plate, to more complex techniques based on scaffolds that better mimic the architecture of the *in vivo* environment. Since its advent there has been some progress in the ways these systems are used in laboratories, nowadays 3D systems have been studied in reproduction in the sense of basic research, to better understand physiology and molecular pathways (Sargus-Patino et al., 2014; Zhao et al., 2015), and also with the objective of applying to obtain better quality gametes and embryos (Kolahi et al., 2012).

Differently from what happens *in vivo*, where embryos develop with the influence of the maternal environment, such as the stiffness and tissue movement, in the laboratory they are produced *in vitro* in two-dimensional cultures. Although we have satisfactory results using conventional IVEP technique, embryos produced *in vitro* are still different from embryos produced *in vivo* (Canovas et al., 2017) and this culminates in a greater pregnancy loss or even problems after birth (Bouillon et al., 2016). In addition, studying embryos after hatching *in vitro* is challenging because of the difficulty of maintaining them, and *in vivo* studies are also complex due to limited access to embryos at this stage. Then, the emergence of new culture techniques that provide better quality embryos or that allow longer development are needed. With this in mind, 3D cultures have great potential to overcome these limitations.

**Figure 2:**
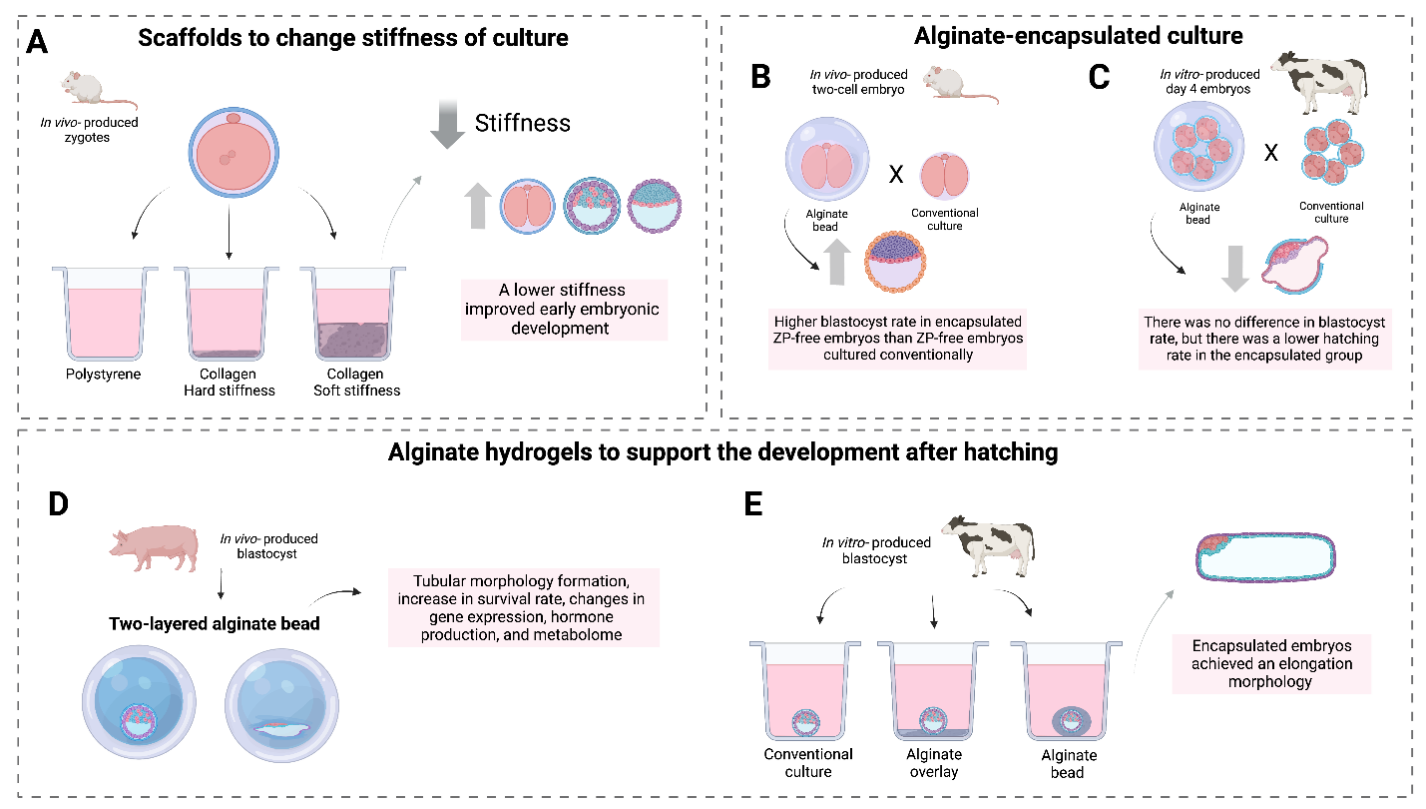
3D cultures applied for embryo production. In (**A**) mouse embryos were produced in a high or low stiffness using collagen scaffolds (Kolahi et al., 2012). In (**B**) mouse embryos without the zona pellucida were developed inside an alginate bead or in conventional culture (Eaton et al., 1990). In (**C**) bovine embryos were cultured in groups of 5 encapsulated inside an alginate bead or in conventional culture (Yániz et al., 2002). In (**D**) porcine blastocysts were cultured in a two-layered alginate bead to support its development after hatching (Sargus-Patino et al., 2014; Laughlin et al., 2017; Walsh et al., 2023). In (**E**) bovine blastocysts in the hatching stage were cultured in an alginate bead or alginate overlay (Zhao et al., 2015). Figure made with Biorender.

When compared to conventional 2D culture, a system created using type I Collagen of a soft stiffness (1 KPa) increased rates of mouse cleavage, blastocyst development, blastocyst hatching, total cell number, trophectoderm cell number, and placental weight after embryo transfer. However, a type I Collagen system created with a harder stiffness decreased these rates (Kolahi et al., 2012) (Figure 2A). Collagen is a natural component of uterine ECM (Lopez-Garcia et al., 2010), so it can mimic uterus elasticity depending on its concentration. The fact that they obtained better results in embryos developed on a soft collagen system than on a hard collagen system, suggests that the embryos are affected by the environment stiffness, and not only by its components. Such findings were even more evident when the experiment was repeated removing the embryo’s zona pellucida. These findings emphasize that embryos perceive the physical signs of the environment and that 3D cultures can be a solution compared to traditional cultures (Kolahi et al., 2012).

An earlier report reinforces the beneficial effects of 3D systems on the mechanical pressure naturally exerted by the zona pellucida. They found that zona pellucida free embryos cultured in a 3.5% alginate hydrogel resulted in higher blastocyst rates than zona pellucida free embryos cultured conventionally (Eaton et al., 1990) (Figure 2B). Alginate is a natural polymer from brown algae, and it can form a stable hydrogel when ionically crosslinked (Lee and Mooney, 2012). Alginate hydrogels are known to be very easy to manipulate, biocompatible, and not cytotoxic (Jalayeri et al., 2017; Vanacker et al., 2012). In addition, bovine embryos encapsulated with 1.5% alginate hydrogels and co-cultured with oviductal cells, had no difference in the blastocyst rate compared to conventional IVEP, however, it presented a reduction in the hatching rate (Yániz et al., 2002) (Figure 2C). When bovine embryos were produced using a super hydrophobic 3D system named liquid marbles, a negative effect on blastocyst development was observed, with changes in DNA global methylation and hydroxymethylation patterns (Ferronato et al., 2023). It suggests that under these experimental conditions, the mechanical force exerted on embryos may not be positive at this stage of development. On the other hand, there are a series of studies that uses 3D cultures to develop embryos after hatching to study the mechanisms of elongation.

Day 9 porcine embryos were double-layered with 0.7% alginate hydrogels and cultured for 96h, interestingly, some of the encapsulated embryos had a tubular morphology, without changes on survival rates, showing that the hydrogel matrix can support embryo elongation through mechanical forces (Sargus-Patino et al., 2014) (Figure 2D). Furthermore, for tubular embryos the expression of steroidogenic genes and estradiol production was more similar to patterns found *in vivo* than in conventional culture *in vitro* or in embryos encapsulated but that did not change the morphology (Sargus-Patino et al., 2014). The same research group conjugated alginate hydrogels with Arg-Gly-Asp and it increased the embryo survival and induced morphological changes, when compared to the normal alginate hydrogel (Laughlin et al., 2017). This model was recently used to study metabolome changes during the elongation process, which identified changes in secreted metabolites that may be correlated with the elongation mechanism (Walsh et al., 2023).

To support bovine embryo development *in vitro* after hatching, day 8 blastocysts were cultured on top or inside 1.5% alginate hydrogels (Zhao et al., 2015). On day 18 encapsulated embryos presented an elongated morphology. After removing them from the hydrogel, embryos adhered to the plate and continued cell proliferation until day 32. In addition, a large number of binuclear cells were found in these embryos, a trophoblast cell subtype, demonstrating that after alginate culture there was also support for cell differentiation (Zhao et al., 2015) (Figure 2E). In humans, Matrigel was used to create a 3D environment for embryo culture to study pre-gastrulation (Xiang et al., 2020a).

The application of 3D cultures in embryonic research can advance the *in vitro* development of embryos and provide insights into the physiological mechanisms of embryo elongation and implantation. In parallel, significant endeavors are being made to establish 3D endometrial cell cultures to enable embryo co-cultures and explore the mechanisms of maternal-embryo communication and elongation, with the goal of producing embryos. Wang et al. used fibrin-agarose as matrix scaffolds to create a human endometrium model (Wang et al., 2012). Epithelial cells formed a monolayer on top of the matrix, and stromal cells migrated into it. The epithelial cells exhibited the ability to spontaneously form glands *in vitro*, while trophoblast-like cell spheroids cultured on top of this system were able to adhere and secrete hCG into the medium. MacKintosh et al. used an electrospun scaffold to create a bovine endometrium model, in which stromal and epithelial cells adhered and proliferated while remaining functional and responsive to hormone and immune stimuli (MacKintosh et al., 2015). In a different approach, trophoblast cell-derived blastocyst-like spheroids were co-cultured with human endometrial stromal cells (HESC) using Matrigel (You et al., 2019). HESC promoted the migration and invasion of trophoblast cells, which was not observed when the cells were epithelial or when Matrigel was used alone. A recent study used a porous alginate scaffold for 3D culture of human epithelial endometrial cells, in which cells remained viable and hormone-responsive for three weeks under hormonal treatment, creating a receptive and non-receptive environment for embryo implantation. This model could potentially use patients' cells to investigate individual mechanisms that may lead to pregnancy failure and enable personalized therapies (Stern-Tal et al., 2020).

Although 3D systems have revolutionized cell culture, they face obstacles in becoming a routine for embryo culture. Currently, in contrast to the standardized protocols for two-dimensional cultures, there is a lack of well-established protocols for embryo culture in 3D systems. Specific modifications to the traditional culture medium, as demonstrated by Xiang et al., are necessary to achieve optimal results in 3D systems (Xiang et al., 2020b). Other variables, such as gaseous diffusion and mechanical force exerted on embryos, also significantly influence development. Despite advances in science that have led to the emergence of new technologies and culture systems, further research is necessary before 3D cultures can be established for *in vitro* embryo culture.

## Organs-on-a-chip and bioprinting

Leveraging 3D printing and microfluidics technologies, organs-on-a-chip can be rapidly designed and optimized for studying mammalian organ-specific physiology, leading to improved *in vitro* organ models for examining aspects of physiology, disease, and toxicology (Huh et al., 2011). Organs-on-a-chip, highlighted by the World Economic Forum as one of the “Top Ten Emerging Technologies” in 2016, offer exceptional control over the culture microenvironment, enabling a direct examination of genetic and environmental factors on cellular function and communication. In the realm of reproductive research, organ-on-a-chip models have been employed to explore areas such as follicle development (Laronda et al., 2017; Xiao et al., 2017), menstrual cycle dynamics (Xiao et al., 2017), embryo-maternal interactions (Ferraz et al., 2018b, 2017), and cancer (Ferraz et al., 2020b).

Organ-on-a-chip technology offers significant potential for process automation in ARTs, due to its integration and adaptability. To develop a more biomimetic system for enhancing fertilization rates and *in vitro* embryo quality, a microfluidic device that mimics the bovine oviduct epithelium was created (Ferraz et al., 2018b, 2017). Bovine oviductal epithelial cells (BOECs) were cultured on this device, forming a tight cell monolayer consisting of ciliated and secretory cells with villus-like structures, like the *in vivo* oviduct environment. The model tested three hormone treatments to simulate the hormonal fluctuations experienced by the oviduct (no hormone, luteal, and pre-ovulatory phases). These treatments influenced both the epithelial barrier, as confirmed by trans-epithelial electrical resistance measurements, and the transcriptome profiles of the BOECs. As seen in *in vivo* BOECs, the pre-ovulatory phase showed increased expression of genes related to ciliogenesis, cilia movement, immune response, and other physiological processes, while the luteal phase exhibited increased cell-cell junction organization, growth factor response, and antioxidant activity (Ferraz et al., 2018b).

This oviduct-on-a-chip model demonstrates potential for more accurate *in vitro* studies of reproductive processes, as the cultured BOECs expressed genes related to sperm-oviduct adhesion, cumulus-oocyte complex-oviduct interaction, fertilization, and embryo development. The device was successfully used for a complete IVF procedure, with a reduced occurrence of polyspermy (Ferraz et al., 2017). However, on-chip fertilization and culture were less successful than an optimized *in vitro* embryo production protocol (Ferraz et al., 2018b). The reduced success can be attributed to nearly half of the mature oocytes/embryos escaping through the pillars, and the shear stress on the embryos. Despite these challenges, the interaction between gametes and zygotes with the epithelium in the oviduct-on-a-chip platform overcomes the changes to the abnormal (de)methylation process that results during standard IVEP (Ferraz et al., 2018b). By comparing the transcriptome of individual bovine zygotes produced under different conditions, the study found that the oviduct epithelium plays an important role in regulating embryo development, since the oviduct-on-a-chip platform successfully rescued the gene expression pattern in half of the analyzed zygotes, highlighting its potential for improving ARTs.

Microfluidics has also been used to create more complex models of the endometrium. In cows, an endometrium-on-a-chip was developed using both epithelial and stromal cells and exposed to varying glucose and insulin concentrations, resulting in changes to the cellular transcriptome and secreted proteome (De Bem et al., 2021). In humans, a vascularized endometrium-on-a-chip was created with five microchannels for 3D culture of stromal fibroblast and endothelial cells, as well as epithelial cells, resulting in a three-layer model that accurately recapitulated *in vivo* endometrial vasculo-angiogenesis and hormonal responses (Ahn et al., 2021). The model was used to evaluate the effects of the emergency contraceptive drug levonorgestrel and as a proof of concept for embryo implantation, providing insights into their underlying molecular and cellular mechanisms (Ahn et al., 2021). In another approach, a multi-organ-on-a-chip platform was employed to culture *ex vivo* mouse ovarian tissue under dynamic microfluidic conditions. This system successfully replicated the human 28-day menstrual cycle, resulting in the ovulation of fertile oocytes (Xiao et al., 2017).

Microfluidic technology has also emerged as a promising approach for generating germ cells from *ex vivo* gonadal tissues. Ovarian follicles are typically encapsulated within a hydrogel matrix for *in vitro* culture and maturation of oocytes, mimicking the natural physical environment of ovarian tissue (Gargus et al., 2020). This method has been successfully applied to a variety of species, including laboratory animals, humans, livestock, dogs, and cats. Recently, individual human follicles were cultured under well-defined dynamic conditions in a microfluidic chamber after being encapsulated in alginate beads (Aziz et al., 2017). This encapsulation process can be scaled down to the single-follicle level and more precisely controlled using droplet microfluidics (Choi et al., 2014), a technique widely used for isolating individual cells within hydrogel microbeads (Kamperman et al., 2018). Regarding the male, mouse prepubertal testicular tissues were viably cultured for up to six months using microfluidic technology, achieving complete gametogenesis. A vascular-like system ensured consistent oxygenation, nutrition, and hormonal stimulation of the seminiferous tubules, ultimately yielding sperm cells capable of embryo development after oocyte injection (Komeya et al., 2016; Yamanaka et al., 2018).

As a step further, 3D printing technology has opened new opportunities for the development of biomimetic and personalized materials. 3D printing technology offers precise control over bulk geometry and internal pore architecture, allowing for sophisticated biomimicry and personalization in the fabrication of materials (Fullerton et al., 2014). 3D printing can be used to create biological scaffolds, which are cell-free structures that can be seeded with cells, or 3D bioprinted structures, which are created by depositing cell-laden “bioinks” in precise 3D locations (Murphy and Atala, 2014). In the reproductive biology field, bio-printed models of testis and ovaries were developed. Using 3D coaxial bioprinting technology, tubular structures resembling the seminiferous tubule were successfully created from single cells obtained from a human testicular biopsy of a donor with non-obstructive azoospermia (Robinson et al., 2022). The cells were first dissociated into single cells, expanded *in vitro* and coaxial bioprinted into tubular structures using an alginate bioink. The coaxial bioprinting allows for the simultaneous extrusion of two bioinks through a coaxial nozzle, here a core bioink is surrounded by a shell bioink, creating a double-layered tubular structure with precise control of the internal architecture and mechanical properties (Robinson et al., 2022). This model allowed for high viability of testicular cells while preserving the main somatic phenotypes of the testicular tissue, with a significant increase in germ cell markers within the 3D bioprinted tubules after 12 days of *in vitro* culture (Robinson et al., 2022).

Another example is the bioprosthetic ovary, in which murine follicles were seeded into 3D-printed gelatin scaffolds (with a tortuous network of interconnected pores), maintained the 3D architecture, survival, and function of the follicles, specifically the hormone production (Laronda et al., 2017). As 3D printing technology continues to develop and improve, it holds great potential in creating personalized scaffolds with customized cell-specific niches, aiding in the development of clinical solutions for patients, better development of IVEP embryos and the study of *in vivo* physiology.

## Advancements, challenges, and potential use in IVEP

Microfluidics has emerged as a powerful technology in IVEP, offering numerous advantages in gamete handling, IVF, and embryo culture. The precise control of fluid dynamics enables the isolation and selection of high-quality gametes, sperm sorting and capacitation, and the optimization of oocyte maturation conditions. Microfluidic platforms have also been employed to improve IVF outcomes by enhancing gamete interactions, reducing polyspermy rates, and improving fertilization efficiency.

Future studies should consider including temperature control elements, such as integrated heaters and temperature sensors, to maintain stable culture conditions; and integrated optical or electrochemical sensors for non-invasive assessment of embryo development and metabolic activity. On-chip embryo culture and monitoring systems can also be combined with automated image analysis and machine learning algorithms to enable high-throughput screening and selection of embryos based on morphological and developmental parameters. This integrated approach has the potential to significantly improve the efficiency and effectiveness of IVEP by providing real-time feedback on embryo development and enabling more informed decision-making during embryo selection. Despite the promising applications of microfluidics in IVEP, several challenges and limitations remain. Integration with existing IVEP workflows, standardization, scalability, biocompatibility, and material-related issues are some of the key obstacles that need to be addressed to fully exploit the potential of microfluidics in IVEP.

3D culture systems have gained significant interest in IVEP due to their ability to mimic the *in vivo* ECM (Baker and Chen, 2012). By providing a more physiologically relevant environment for embryo development, 3D culture systems have been shown to improve cell-to-cell and cell-to-ECM interactions, which could result in enhanced embryo development and quality. These systems also provide a platform for investigating embryo-maternal communication, as they enable the study of trophoblast invasion, modeling of embryo implantation, and exploration of the role of paracrine signaling (Kagawa et al., 2022; Park et al., 2022). Understanding these processes is crucial for optimizing IVEP outcomes and for gaining deeper insights into early embryonic development. However, the application of 3D culture systems in IVEP is not without challenges. Standardization, reproducibility, integration with current IVEP methods, and ethical and regulatory considerations are some of the key issues that need to be addressed for 3D culture systems to reach their full potential in IVEP.

**Figure 3:**
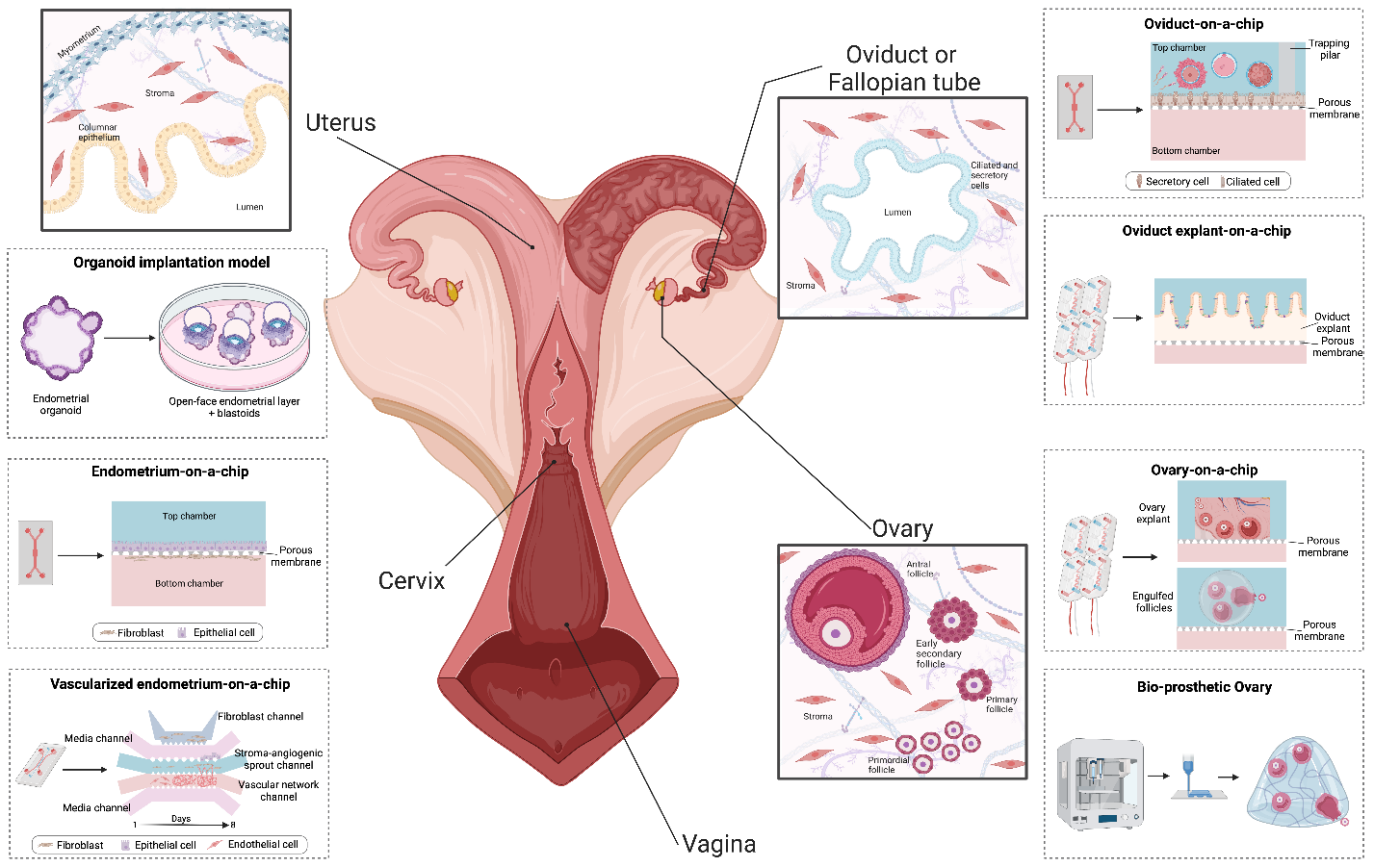
Organs-on-a-chip models of female reproductive tissues. Figure depicts uterine (Ahn et al., 2021; De Bem et al., 2021; Kagawa et al., 2022), oviductal (Ferraz et al., 2018b; Xiao et al., 2017) and ovarian (Laronda et al., 2017; Xiao et al., 2017) tissues and its respective models that combine 3D culture, microfluidic and/or bio-printing to develop more bio-mimetic *in vitro* models. Figure made with Biorender.

Finally, the integration of microfluidics, 3D culture systems and bioprinting offers the potential to revolutionize IVEP by combining the advantages of these technologies. By creating a more biomimetic environment with precise control over culture (Wale and Gardner, 2016), the combined approach has the potential to improve embryo development and quality. The integration of these technologies was already used to create endometrial (Ahn et al., 2021; De Bem et al., 2021; Xiao et al., 2017) and oviductal (Ferraz et al., 2020b; Ferraz et al., 2018b, 2017; Xiao et al., 2017; Zha et al., 2023) models which can provide valuable insights into embryo-maternal communication and also to recapitulate follicle growth and ovulation *in vitro* (Laronda et al., 2017; Xiao et al., 2017) (Figure 3). However, several challenges remain, including technical and engineering complexities, standardization and reproducibility issues, and the need for validation against conventional IVEP methods. Addressing these challenges will be crucial for the successful implementation of integrated microfluidics and 3D culture systems in IVEP and their widespread adoption in reproductive medicine and research. Nevertheless, the integration of these cutting-edge technologies is expected to pave the way for significant progress in reproductive medicine and embryology, ultimately benefiting researchers, patients and society as a whole.
